# Supplementary material for: Direct detection of SABRE-SHEATH hyperpolarization and spin-lattice relaxation of [1-13C]pyruvate
Source: Commun Chem. 2025 Dec 20;9:44. doi: 10.1038/s42004-025-01851-1 (PMC12827285; doi:10.1038/s42004-025-01851-1)
Supplement: Supplementary file 2 — Supplementary Information [file 42004_2025_1851_MOESM2_ESM.pdf]

Supplemental Information

John Z. Myers[1], Markus Plaumann[2], Kai Buckenmaier[3], Andrey N. Pravdivtsev[4], and Rainer Körber[1]

[1]Physikalisch-Technische Bundesanstalt, Berlin
[2]Otto-von-Guericke University, Magdeburg
[3]Max Planck Institute for Biological Cybernetics, Tübingen
[4]University Hospital Schleswig-Holstein and Kiel University

Supplementary Note 1 Sample Loss over Time

As stated in Section 4.3.2, the NMR data used for characterizing T_B and T_1 were corrected for sample loss over experimental time. During the characterization of T_B and T_1, control measurements were regularly performed under the following conditions: ambient temperature (22°C) and pressure, $B_\mathrm{Hyp} = 500$ nT (z-axis), 40 s bubbling time, 40 s acquisition at 20 kHz and $B_\mathrm{Det} = 38.6$ μT (x-axis). The data were handled as described in Section 4.3.1. For the control measurements, the summed amplitude of the ^{13}C signals were normalized against the maximum of the summed amplitudes among the control measurements. This is shown in Figure S1. For the acquisitions used to characterize T_B and T_1, they are also shown in Figure S1, where the y-value was determined, using linear interpolation between the closest control measurements. The data were then corrected for sample loss by dividing the amplitudes of the Lorentzian fits of the ^{13}C signals by the proportion expected to be achieved of the maximum possible signal when there has been no sample loss (the y-values from Figure S1).

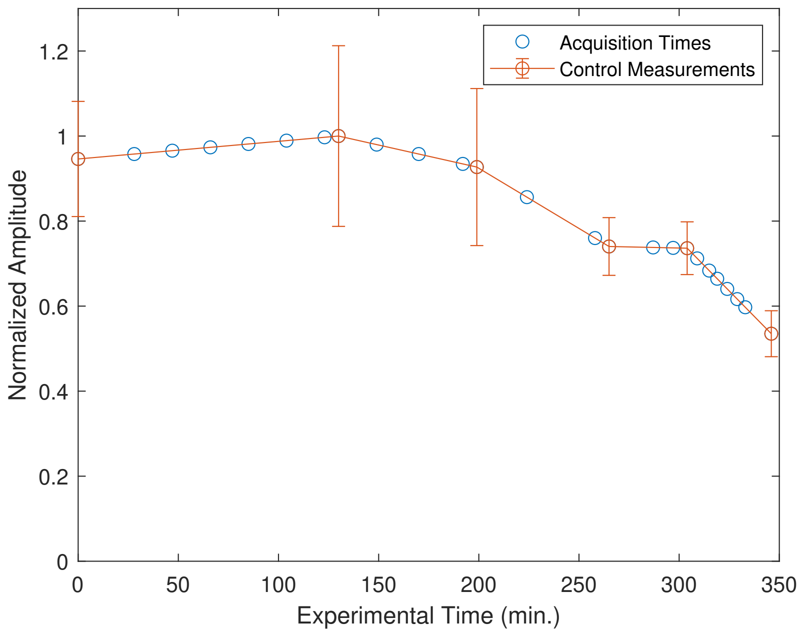

Figure S1: **Sample Loss over Experimental Time.** For the control measurements, the summed amplitudes of the ^{13}C signals from [1-^{13}C]pyruvate hyperpolarized via SABRE-SHEATH are normalized relative to the largest control measurement and plotted against the time the acquisition was performed relative to the first control measurement. The times all of the acquisitions for the characterization of T_B and T_1 were performed are also shown, where the y-value is given by linear interpolation between the two closest control measurements.

Supplementary Note 2 System Setup

A picture of the setup is shown in Figure S2.

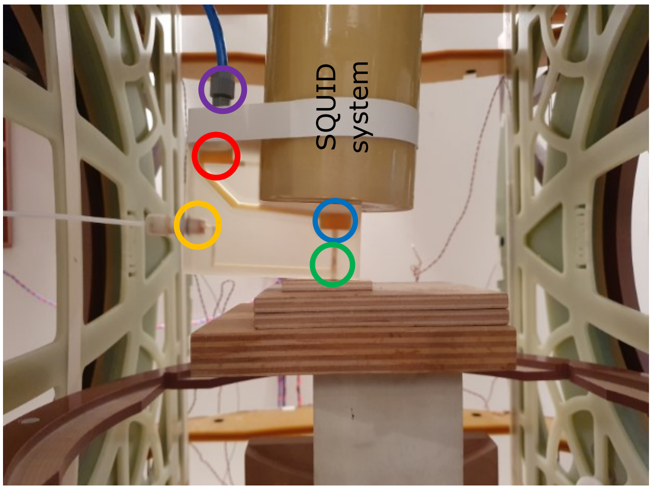

Figure S2: **Photo of the Experimental Setup.** pH_2 gas enters the SABRE reactor through the reactor inlet circled in yellow. The gas then travels through a static Kenics mixer circled in green mixing with the [1-^{13}C]pyruvate sample. The main sample volume is circled in blue. This part of the SABRE reactor is directly underneath the pickup coil of the SQUID detector, which is housed inside of the dewar. Circled in red is the sample reservoir, which allows for maintaining a full sample volume as sample is displaced by pH_2 gas and as the sample evaporates into the environment. The pH_2 outlet is shown in purple. This photo is adapted from Figure 2b from Myers *et al.* [1].

Supplementary Note 3 Fitting Equations and Parameters

The generalized fitting equation used for determining T_B is given in Eq. (S1), where A is the fitted amplitude, max the maximum amplitude from the data, C a multiplicative fitting constant (theoretically equivalent to max), t the pH_2 bubbling time and delay the offset time from where the fit was applied. Due to not being sensitive enough to detect the full ^{13}C quadruplet without hyperpolarization, the delay parameter of the fit was necessary, since it was not possible to fit from $t = 0$.

$$A = \mathrm{max} - C \exp\left[-\frac{1}{T_\mathrm{B}}\left(t - \mathrm{delay}\right)\right] \tag{S1}$$

The generalized fitting equation used for determining T_1 is given in Eq. (S2), where C should also correspond to the maximum amplitude. delay was also necessary here, due to performing the fit only after the impact of effects likely originating from fluid dynamics were no longer relevant to the model.

$$A = C \exp\left[-\frac{1}{T_1}\left(t - \mathrm{delay}\right)\right] \tag{S2}$$

All fitting parameters relevant to the results quoted in Section 2 with their uncertainties are given in Table S1.

Supplementary Note 4 Vibrational Noise versus B_Hyp

The effect of generating the B_Hyp field on the detected vibrational noise in the direct detection setup is shown in Figure S3. The effect is roughly proportional to the strength of the B_Hyp field, evidenced by the peak to peak amplitude at a B_Hyp of 500 nT being ~0.6 pT versus ~0.18 pT for a B_Hyp of 150 nT.

	C (pT)	95% CI (pT)	T_B or T_1 (s)	95% CI (s)	delay (s)	max (pT)
**Buildup (NMR)**	10.9	[10.54, 11.25]	37.0	[33.5, 41.4]	4	11.0
**Buildup no last point (NMR)**	9.3	[8.6, 10.0]	26.0	[21.0, 34.2]	4	9.3
**Relaxation (NMR)**	21.3	[20.2, 22.3]	43.3	[38.2, 50.2]	10	–
**Buildup S1 500 nT (direct)**	8.8	[8.8, 8.8]	25.7	[25.7, 25.7]	5	8.6
**Relaxation S1 500 nT (direct)**	9.0	[9.0, 9.0]	42.0	[42.0, 42.0]	5	–
**Buildup S2 500 nT (direct)**	3.9	[3.9, 3.9]	25.6	[25.6, 25.6]	5	4.4
**Relaxation S2 500 nT (direct)**	4.7	[4.7, 4.7]	43.4	[43.4, 43.4]	5	–
**Buildup S2 150 nT (direct)**	1.8	[1.8, 1.8]	35.9	[35.9, 35.9]	5	2
**Relaxation S2 150 nT (direct)**	2.1	[2.1, 2.1]	40.1	[40.1, 40.1]	5	–

Table S1: Values of fitting parameters.

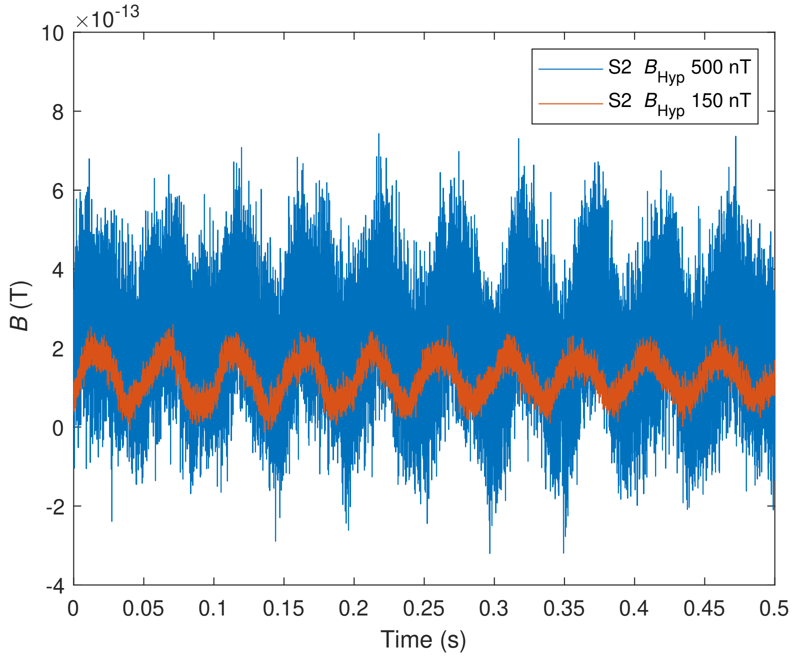

Figure S3: **Effect of $B_{\mathbf{Hyp}}$ on Vibrational Noise.** This is shown over a portion of a single relaxation cycle for both B_{Hyp} of 150 nT and 500 nT.

References

[1] J. Z. Myers, F. Bullinger, N. Kempf, M. Plaumann, A. Ortmeier, T. Theis, P. Povolni, J. Romanowski, J. Engelmann, K. Scheffler, *et al.*, “Zero to ultralow magnetic field NMR of [1-^{13}C] pyruvate and [2-^{13}C] pyruvate enabled by SQUID sensors and hyperpolarization,” *Physical Review B*, vol. 109, no. 18, p. 184443, 2024.
